# Retraction: Public perceptions About Foreign Investment, A PLS-SEM Analysis toward republic sustainable infrastructure

**DOI:** 10.1371/journal.pone.0309791

**Published:** 2024-08-28

**Authors:** The PLOS ONE Editors

After this article [1] was published, concerns were identified about the integrity of the peer review process.

A member of the *PLOS ONE* Editorial Board who was not involved in the article’s pre-publication peer review reviewed the article and noted concerns including inadequate and inaccurate referencing; insufficient rationale for hypotheses; insufficient reporting of the methodology that prevents reproducibility and interpretation; and statements and conclusions that are not supported by the study design and results. The *PLOS ONE* Editors concluded that the article does not meet the journal’s criteria for publication.

In light of the above concerns, the *PLOS ONE* Editors retract this article. We regret that the issues were not identified prior to the article’s publication.

The authors did not agree with the retraction.
